# Elevated urinary level of vitamin D-binding protein as a novel biomarker for diabetic nephropathy

**DOI:** 10.3892/etm.2013.1426

**Published:** 2013-11-26

**Authors:** XIAO-QIN TIAN, LI-MIN ZHAO, JIA-PU GE, YAN ZHANG, YAN-CHENG XU

**Affiliations:** 1 Department of Endocrinology, Zhongnan Hospital, Wuhan University, Wuhan, Hubei 430071, P.R. China; 2 Department of Endocrinology, People’s Hospital of Xinjiang Uygur Autonomous Region, Urumqi, Xinjiang 830011, P.R. China; 3 Department of Endocrinology, Traditional Chinese Medicine Hospital, Xinjiang Medical University, Urumqi, Xinjiang 830000, P.R. China

**Keywords:** vitamin D binding protein, urine, diabetic nephropathy

## Abstract

Improving the early prediction and detection of diabetic nephropathy (DN) remains a great challenge in disease management. The aim of this study was to evaluate the early detection power of urinary vitamin D-binding protein (VDBP) for the diagnosis of DN. Urine samples were obtained from 45 healthy volunteers and 105 diabetic patients with normoalbuminuria (DM group), microalbuminuria (DN1 group) and macroalbuminuria (DN2 group) (n=35 per group). The VDBP expression patterns in urine from patients and controls were quantified by western blot analysis. The excretion levels of urinary VDBP were quantified with enzyme-linked immunosorbent assay. The quantification results were obtained by correcting for creatinine expression and showed that urinary VDBP levels were significantly elevated in the patients of the DN1 and DN2 groups compared with those of the DM group and normal controls (1,011.33±325.30 and 1,406.34±239.66 compared with 466.54±213.63 and 125.48±98.27 ng/mg, respectively) (P<0.001). Receiver operating characteristic analysis of urinary VDBP levels for the diagnosis of DN rendered an optimum cut-off value of 552.243 ng/mg corresponding to 92.86% sensitivity and 85.00% specificity, which also showed an area under the ROC curve of 0.966. In conclusion, the findings of the present study suggest that urinary VDBP may be a potential biomarker for the early detection and prevention of DN. Further studies are required to examine the pathogenic mechanisms of elevated VDBP levels and their role in the diagnosis of DN.

## Introduction

Diabetes is the fifth leading cause of mortality in the United States and the world prevalence of diabetes is increasing at an alarming rate with >1 million new patients per year diagnosed in the United States (1,2). Furthermore, diabetes results in substantial mortality and morbidity (3). Although the current methods of diagnosis and treatment of diabetes have improved, the long-term prognosis remains poor (4). In addition, there are few effective preventive measures against its development. Epidemiological data suggested that disease prevalence is likely to continue to increase globally without effective prevention and control (5).

Diabetic nephropathy (DN) is one of the most common complications, which results in chronic kidney disease in diabetic patients. It is also one of the causes of increased cardiovascular mortality (4). However, a considerable amount of significant diabetic renal structural injury may occur in absolute clinical silence, which renders diagnosis difficult. Currently, routine kidney biopsy for DN is not permitted in clinical practice, as the procedure is invasive. Microalbuminuria is considered as the best available, non-invasive marker for DN risk, but certain studies have shown it to have inadequate specificity and sensitivity (6,7). Thus, additional studies into novel invasive risk markers are required, and feasible measures for the diagnosis of DN prior to advanced renal dysfunction are considered to be of clinical importance with public health implications.

Vitamin D-binding protein (VDBP), also known as gc-globulin, bonds and transports vitamin D throughout the body. It also is important in the actin-scavenger system, ensuing immune responses and inflammation processes (8). Clinically, it has been demonstrated that exaggerated excretion of urinary VDBP is associated with tubular dysfunction (9). Therefore, it was hypothesized that the loss of urinary VDBP is likely to be elevated in diabetic patients and particularly accentuated in those patients with DN. In the present study, the early detection and predictive value of urinary VDBP for DN was assessed.

## Materials and methods

### Patient selection and urine specimen collection

In this study, 105 Chinese Han individuals with diabetes and 45 healthy volunteers (control group) were recruited from the Traditional Chinese Medicine Hospital attached to Xinjiang Medical University (Urumqi, China) from June 2012 to January 2013. The patients were divided into three groups according to the value of the urinary albumin:creatinine (Cr) ratio (UACR): DM group without nephropathy and albuminuria (UACR<30 mg/g, n=35), early DN group (DN1) with microalbuminuria (30≤UACR<300 mg/g, n=35) and overt DN group (DN2) with macroalbuminuria (UACR≥300 mg/g, n=35). All study subjects were >18 years old and blood pressure was maintained at 140/90 mmHg by the use of angiotensin-receptor blockers or angiotensin-converting enzyme inhibitors. Moreover, blood glucose was controlled with human insulin. Within the 2 weeks prior to urine collection, patients did not receive additional medicine and the controls did not receive any medication. Patients were excluded if they showed the following: Liver diseases, autoimmune diseases, inflammatory diseases, pregnancy, urinary system disorders, tumors, infections, decompensated heart failure, cardiovascular events within 6 months, hematological diseases and known renal diseases other than DN. Studies were approved by the local ethics committee of the Traditional Chinese Medicine Hospital attached to Xinjiang Medical University (Urumqi, China). All patients were informed about the purpose of the study and gave their written consent.

### Urine specimen collection

The second voided clean-catch urine samples from all patients were collected early in the morning. Each urine sample (20 ml) was directly collected into a sterile plastic tube and then immediately centrifuged at 2,500 × g for 10 min at 4°C to remove cell debris and particulate matter. The supernatant was stored at −80°C for further analysis. Repeated freeze-thaw cycles were avoided. Clinical data of all patients were also collected.

### Western blot analysis

The stored supernatant was used for western blot analysis after measuring the protein concentrations. The urine samples were first thawed on ice, adding 1 mmol/l phenylmethanesulfonyl fluoride (Sigma, St. Louis, MO, USA), and then centrifuged using Centricon Plus-20, 10,000 MWCO devices (Millipore, Bedford, MA, USA). Then the concentrated urinary proteins were precipitated using a ReadyPrep 2-D clean up kit (GE Healthcare, Piscataway, NJ, USA) to remove other interfering components according to the manufacturer’s instructions The concentrations of urinary proteins were determined by using the Bradford protein assay kits (GE Healthcare). The samples examined were from patients from the DM, DN1, DN2 and control groups (n=8 per group). A total of 20 μg prepared proteins isolated from the urine samples were electrophoresed on a 12% sodium dodecyl sulfate-polyacrylamide gel. The proteins were then transferred onto polyvinylidene fluoride membranes (Immobilon P; Millipore, Billerica, MA, USA). The membranes were blocked for 1 h at 37°C in a solution of TBS containing 5% non-fat milk powder and 0.1% Tween-20 (TBS-T) and then incubated overnight at 4°C with rabbit monoclonal primary antibody against human VDBP (diluted 1:1,000; Abcam, Cambridge, UK). Membranes were washed three times for 10 min in TBS-T and then incubated with horseradish peroxidase horseradish-coupled goat anti-rabbit IgG (Beijing Zhongshan Biotechnology Co. Ltd., Beijing, China) at a 1:500 dilution at room temperature for 1 h. The proteins were detected using an enhanced chemiluminescence detection system (ECL-Direct systems RPN3000; Pierce Biotechnology, Inc., Rockford, IL, USA). Triplicate gel images of identical samples were used for analysis. It was quantified by strip densitometry.

### Enzyme-linked immunosorbent assay (ELISA)

All the samples were centrifuged at 3,500 × g for 5 min at 4°C to remove interfering matter prior to ELISA analysis. The concentrations of VDBP in the urine samples were measured with a Human Vitamin D BP Quantikine ELISA kit (DVDBP0; R&D Systems, Minneapolis, MN, USA). The assay was performed according to the instructions recommended by the manufacturer. The standard curve was created using the lyophilized human VDBP standard preparation supplied with the assay. Following the colorimetric reaction, the optical density (OD) readings were converted to concentrations in ng/ml based on quantification of the OD at 450 nm using an eight-channel spectrophotometer (Fast model; BD Biosciences, Franklin Lakes, NJ, USA). Measured VDBP levels ranged from 0 to 250 ng/ml. Urine Cr levels were measured at the Department of Clinical Laboratory, Traditional Chinese Medicine Hospital attached to Xinjiang Medical University (Urumqi, China). The levels of VDBP were normalized according to urine Cr concentrations so as to avoid the influence of urine volume and presented as VDBP:Cr ratio (VDBP-Cr; ng/mg of Cr) (10). Every sample was tested in duplicate.

### Statistical analysis

All data were collected and presented as the mean ± standard deviation. The differences among groups were compared with Student’s t-test between two groups or one-way analysis of variance for three groups. Receiving operating curve (ROC) analyses were used to explore the diagnostic performance of urinary VDBP:Cr over a range of possible clinical results on the basis of estimating the sensitivity versus its false-positive rate at optimal cut-offs (10,11). The best statistical cut-off value of VDBP:Cr was defined, which means the point at which the sum of sensitivity and specificity is more than that at other points. Pearson’s correlation coefficient analysis was employed to explore the association between the VDBP:Cr and the clinical features of patients. Multivariate logistical regression analysis was utilized to determine the risk factors for DN. All statistical analyses were performed with SPSS software, version 13.0 (SPSS, Chicago, IL, USA). All tests were two tailed and P<0.05 was considered to indicate a statistically significant difference.

## Results

### Clinical characteristics of patients

The clinical characteristics of all patients are shown in Table I. Samples from 35 DM, 35 DN1 and 35 DN2 patients and 45 controls were collected for ELISA analysis. Analysis of the patient data indicated that there were no statistically significant differences in the majority of clinical characteristics (e.g. age, gender, smoking and duration of diabetes) among the four groups. However, significant differences were identified in serum Cr, UACR and systolic blood pressure between certain groups (serum-Cr: DN2 vs. control P=0.025, DN2 vs. DM: P=0.012 DN2 vs. DN1: P=0.034, UACR-DN1 vs. control: P<0.001, DN1 vs. DM: P<0.001; DN2 vs. control P<0.001, DN2 vs. DM: P<0.001, DN2 vs. DN1: P<0.001; systolic blood pressure-DN1 vs. control: P=0.021, DN2 vs. control: P=0.014). Additionally, as the patients in each experimental group had taken similar medications, the effect of taking medicine on the results was ignored.

### Analysis of urinary VDBP by western blotting

**Figure 1:**
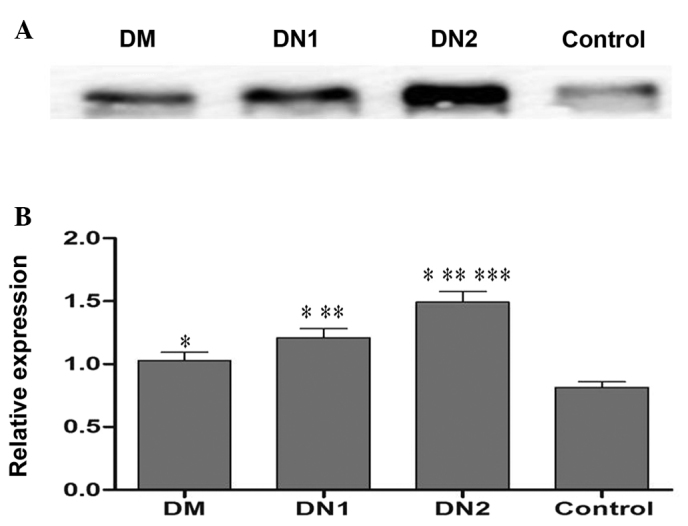
VDBP expression levels were verified in the urine samples. (A) Western blot analysis of VDBP expression in urine samples of DM, DN1, DN2 and control groups. (B) VDBP protein expression levels in individual urine samples was calculated according to the immunosignals quantified by densitometric scanning. Data are expressed as the mean ± standard deviation from three independent experiments. ^*^P<0.05 compared with that of the control group; ^**^P<0.05 compared with that of the DM group and ^***^P<0.05 compared with that of the DN1 group. DM, UACR<30 mg/g; DN, diabetic nephropathy; DN1, with microalbuminuria 30<UACR<300 mg/g; DN2, with microalbuminuria UACR>300 mg/g; UACR, urinary albumin:creatinine ratio; VDBP, vitamin D binding protein; UACR, urine:creatinine ratio.

To verify the expression of urinary VDBP in individual urine samples by western blotting, 32 samples from the DM, DN1, DN2 and control groups (n=8 per group) were randomly selected. The results demonstrated that urinary VDBP levels were significantly upregulated in the DM group compared with that of the control group (Fig. 1). Furthermore, the levels of urinary VDBP were significantly elevated in the DN groups compared with those of the DM and control groups (Fig. 1).

### Detection of urinary VDBP expression

To explore the changes of urinary VDBP expression in DN patients, ELISA analysis was conducted on the 150 urine samples from the DM, DN1, DN2 and control groups.

**Figure 2:**
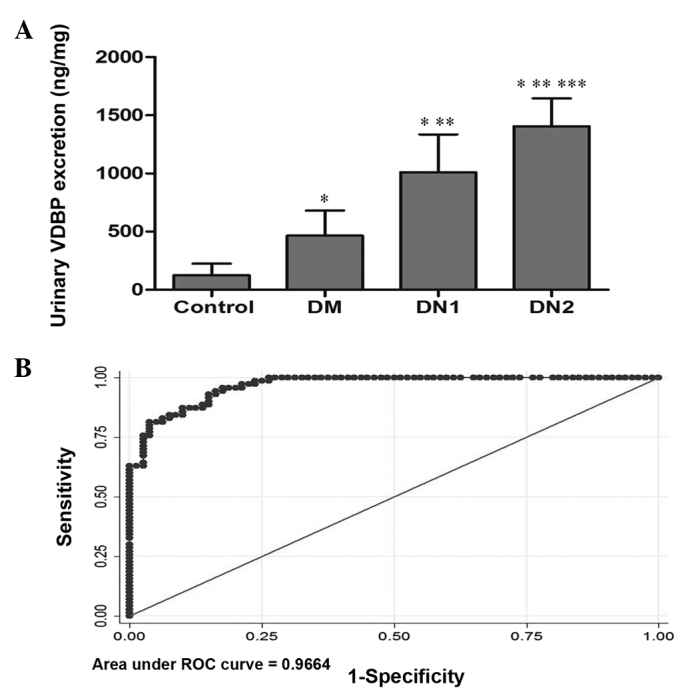
Evaluation of urinary VDBP levels as a biomarker for DN. (A) ELISA quantification of VDBP levels in the urine of DM, DN1, DN2 and control groups. Data are expressed as the mean ± standard deviation. ^*^P<0.05 compared with the control group; ^**^P<0.05 compared with the DM group; and ^***^P<0.001 compared with the DN1 group. (B) ROC curve of urinary VDBP as a biomarker of early detection and prevention for DN was based on an optimum cut-off value of 552.243 ng/mg corresponding to 92.86% sensitivity and 85.00% specificity. The area under the ROC curve was 0.966 (95% CI, 0.924–0.989). VDBP, vitamin D binding protein; DN, diabetes nephropathy; DM, UACR<30 mg/g; DN, diabetic nephropathy; DN1, with microalbuminuria 30<UACR<300 mg/g; DN2, with microalbuminuria UACR>300 mg/g; UACR, urinary albumin:creatinine; ROC, receiver operating characteristics.

The levels of urinary VDBP were significantly higher in patients than in controls (961.41±542.77 versus 125.48±98.27 ng/mg, P<0.001; Fig. 2A). After division of the patients into groups by UACR, it was indicated that the expression levels of urinary VDBP were significantly higher in the DN1 and DN2 groups than in the DM group (1,011.33±325.30 and 1,406.34±239.66 versus 466.54±213.63 ng/mg, respectively; P<0.001). A significant difference was also observed between the DN2 and DN1 groups (P<0.001; Fig. 2A and Table II).

### Correlation and multivariate logistical regression analyses

Additional subgroup analyses were performed to clarify the correlation between urinary VDBP levels and clinical characteristics of the patients (Table II). No significant association was observed between the urinary loss of VDBP and the clinical features of DM patients, such as age, gender, smoking, duration of DM and hypertension, with the exception of DN and renal dysfunction. The enhanced excretion levels of urinary VDBP were significantly higher in patients with renal dysfunction than in the patients without dysfunction (1,263.15±531.33 versus 790.27±473.08 ng/mg; P<0.001). Furthermore, higher urinary VDBP concentrations were detected in the DN1 and DN2 groups than in the DM group (P<0.001). The Spearman rank correlation was 0.707, which indicated that the levels of urinary VDBP had a strong positive correlation with the development of DN. Also, multivariate logistical regression analysis was conducted to assess the independent risk factors for DN. The results suggested that serum Cr may be an independent predictor of DN (OR=1.29, 95% confidence interval, 1.02–1.57; P=0.04).

### Evaluation of urinary VDBP as a biomarker for DN

Following quantitative analysis of 150 urine samples by ELISA, ROC curves were used to assess the potential utility of urinary VDBP detection in patients with DN. The area under the ROC curve of urine VDBP levels for the diagnosis of DN was 0.966 (95% CI, 0.924–0.989). The analysis rendered an optimum cut-off value of 552.243 ng/mg corresponding to 92.86% sensitivity and 85.00% specificity (Fig. 2B).

## Discussion

Improving the early prediction and detection of DN remains a great challenge in disease management (13,14). Improving the predictive ability of testing would greatly benefit the treatment of patients with DN and facilitate the monitoring of the condition. To explore whether urine VDBP levels may be a novel non-invasive biomarker for DN, the results of the present study demonstrated that the expression level of urinary VDBP was highly upregulated in patients with DN. Furthermore, the levels of VDBP were measured in the urine samples from the control, DM and DN groups.

VDBP is a 58-kDa glycoprotein and is present in the serum at a concentration of 300–600 mg/ml (15). It serves as the main carrier protein for vitamin D in the bloodstream. VDBP is important in the bioavailability of active 1,25-dihydroxyvitamin D (1,25(OH)_2_D) and its precursor 25-hydroxyvitamin D (25OHD) (16,17). The transportation of vitamin D by VDBP is important for the function of a wide variety of tissues and changes in VDBP activity result in the development of a number of diseases (8).

In addition to its transport function, VDBP is the parent molecule of VDBP-maf (macrophage activating factor). VDBP-maf is the product of the selectively deglycosylated form of VDBP and has been demonstrated to be a potent antiangiogenic and antitumorigenic molecule (18). Such functions would greatly benefit the regulation of the growth of cancer cells and protection against certain immune disorders (19). These important and diverse properties of VDBP have been suggested in previous studies with regard to a number of tumor types (20,21). Moreover, VDBP is important in the actin scavenger system and inflammation processes (8). Studies concerning the actions of VDBP in the kidney have received increased attention and have suggested that VDBP is vital in the endocrine biosynthetic process of 1,25(OH)_2_D within renal proximal tubules; in this process, 25OHD binds to VDBP and the complex is actively recovered from glomerular filtrate through megalin-mediated receptor endocytosis (22,23).

In the present study, following quantitative measurements of 150 urine samples with ELISA, it was identified that the VDBP expression levels were significantly higher in the urine samples from patients with DN than in the urine from the DM and control groups. Furthermore, a strong positive correlation was observed between the urinary VDBP levels and the development of DN. ROC analysis rendered that an optimum cut-off value of urinary VDBP of 552.243 ng/mg corresponding to 92.86% sensitivity and 85.00% specificity is appropriate for detecting DN. On the basis of these analyses, urinary VDBP was indicated to be a potential biomarker for the early detection and prevention of DN.

The reasons underlying the enhanced excretion of urinary VDBP in patients with DN remain unclear. One possible explanation is that elevated urinary VDBP levels may be associated with renal tubular damage in DN patients (24,25). Renal tubular epithelial cell damage becomes increasingly severe as DN develops. In a previous study, increased excretion of urinary VDBP was observed following long-term cadmium exposure, and it was suggested that the marked loss of VDBP in the urine may be linked to renal tubular dysfunction and bone lesions in the inhabitants of cadmium-polluted areas (26). In addition, it has been demonstrated that the presence of vitamin D deficiency or insufficiency in patients with diabetes is independently associated with the development of DN. Moreover, exaggerated urinary excretion of VDBP was observed in patients with Type I diabetes, which contributed mechanistically to vitamin D deficiency in this disease (9,27,28). Therefore, a further possibility for the elevated urinary VDBP levels identified in the present study may be associated with the relatively lower serum vitamin D levels. Further studies are required to clarify the role of VDBP in the pathogenesis of DN.

Clinically, there have been studies on aspects of other diseases, which demonstrated the increased urinary excretion of VDBP. Recently, Mirković *et al* indicated that the urinary excretion of VDBP may be a novel urinary biomarker of tubulointerstitial damage (29). Cho *et al* demonstrated that the urinary VDBP loss was significantly elevated in patients with endometriosis than those without. However, the authors suggested that urinary VDBP has limited value as a potential early diagnostic biomarker for endometriosis (10). A study by Zoidakis *et al* identified that the reduction of VDBP levels in the urine of patients with invasive bladder cancer was significant (30), which is consistent with the findings by Li *et al*(8). Moreover, Li *et al* also demonstrated that the expression levels of urinary VDBP were positively associated with the pathological classification of bladder cancer (9). Their results suggested that urinary VDBP may be a potential non-invasive biomarker for the early diagnosis and effective surveillance of bladder cancer (8). In the present study, to the best of our knowledge, it was demonstrated for the first time that increased urinary VDBP levels occurred in patients with DN, and there was a strong positive association between urinary VDBP levels and the development of DN.

An important limitation of the present study regarding the specificity of this biomarker should be considered when urinary VDBP detection is used for early prevention of DN. It has been demonstrated that urinary VDBP levels are closely associated with renal dysfunction. In the present study, urine samples were collected from patients with DN only, but not from patients with additional nephropathies. This may have caused an overestimation of the specificity of VDBP as a biomarker for the detection of DN. Therefore, further studies including a larger sample and analyses from patients with various types of non-diabetic nephropathy are required to clarify this issue.

In conclusion, the current study demonstrated that urinary VDBP levels were significantly elevated in patients with DN. Moreover, a strong positive correlation was observed between the expression level of urinary VDBP and the development of DN. Thus, the findings indicate that urinary VDBP levels are a potential biomarker for the early detection and prevention of DN. Further studies are warranted to examine the pathogenic mechanisms of elevated VDBP and its role in the diagnosis of DN.

## Figures and Tables

**Table I:** Clinical and demographic data for the patients subjected to ELISA analysis.

Characteristics	Control	DM group	DN1 group	DN2 group
Cases (n)	45	35	35	35
Age (years)	60.89±13.92	64.63±11.76	64.49±11.78	64.80±12.98
Gender (n)				
Male	31	22	18	21
Female	14	13	17	4
Smoking (n)				
Yes	20	14	17	19
No	25	21	18	16
Diabetes (n)				
Type 1	0	5	5	7
Type 2	0	30	30	28
Diabetic duration (years)	0	11.43±7.83	12.77±8.65	11.57±6.45
Systolic BP (mmHg)	115±8.32	120±9.59	134±8.76*	143±10.32*
Serum creatinine (μmol/l)	99.84±24.23	92.43±36.65	107.34±46.62	142.46±55.10*,**,***
UACR (mg/g)	10.52±2.78	13.08±4.11	134.66±47.2*,**	1603.09±544.60*,**,***
Taking ACEI (n)	0	12	15	14
Taking ARB (n)	0	13	10	12
Insulin therapy (n)	0	35	35	35

Measurement data are presented as the mean ± standard deviation.

* P<0.05 compared with the control group;

** P<0.05 compared with the DM group; and

*** P<0.05 compared with the DN1 group.

DM, UACR<30 mg/g; DN, diabetes nephropathy; DN1, with microalbuminuria 30<UACR<300 mg/g; DN2, with microalbuminuria UACR>300 mg/g; UACR: urinary albumin:creatinine ratio; BP, blood pressure; ACEI, angiotensin-converting enzyme inhibitor; ARB, angiotensin-receptor blocker; ELISA, enzyme-linked immunosorbent assay.

**Table II:** Correlation between levels of urinary VDBP and clinical features of diabetic patients.

Clinical features	No.	Urinary VDBP levels (ng/mg)	P-value
Age			
>65 years	52	1009.52±533.27	0.37
≤65 years	53	914.20±552.91	
Gender			
Female	44	1001.44±491.33	0.52
Male	61	932.53±579.32	
Hypertension			
With	36	1050.86±426.16	0.22
Without	69	914.74±592.15	
Smoking			
Yes	50	1043.62±618.68	0.14
No	55	886.66±456.19	
Renal dysfunction			
With	38	1263.15±531.33	<0.001
Without	67	790.27±473.08	
Diabetes			
Type 1	18	1083.46±582.21	0.30
Type 2	87	936.15±534.31	
Diabetic duration			
>12 years	43	975.74±543.32	0.82
≤12 years	62	951.46±546.60	
DM group	35	466.54±213.63	<0.001
DN1 group	35	1011.33±325.30	
DN2 group	35	1406.34±539.66	

DM, UACR<30 mg/g; DN, diabetic nephropathy; DN1, early DN with microalbuminuria (30<UACR<300 mg/g); DN2, overt DN group with macroalbuminuria (UACR>300 mg/g). UACR, urinary albumin:creatinine ratio; VDBP, vitamin D binding protein.
